# Practical Notes and Formulæ

**Published:** 1881-11-20

**Authors:** 


### PRACTICAL NOTES AND FORMULÆ.

**The Use of Quinine.**—I see in No. 15, current volume, where S. S. F., of Pa., puts some queries that have not been answered. 1st. To disguise the bitter taste of sulph. quinine entirely, is next to impossible, but here are three methods which greatly diminish its bitterness—

℞ Quiniæ sulph.............................. gr. xij.
Acidi tartarici.............................. gr. vj.
Syrup aurantii corticis........................ f. ℥ij.
M. Sig. One to two teaspoonfuls.

℞ Quiniæ sulph.............................. gr. xij,
Syrup acaciæ.............................. } aa f. ℥iss.
Aqua cinnamomi.......................... }
M. Sig. Shake well. Dose, one to two teaspoonfuls, followed by a hearty draught of water. Is nearly tasteless, the quinia salt being in suspension, not in solution.

℞ Quiniæ sulph.............................. gr. xij,
Syrup sarsap. comp.......................... ℥iss,
Acidi tannici.............................. gr. ij.
M. Sig. Dose, one to two teaspoonfuls.

Now, in regard to preventing relapses in intermittent fever or ague, my experience is this: There is no known remedy, unless taken continuously for some time, that will prevent a return of the paroxysms: that is, the patient still remaining in a malarious locality. But S. S. F. will find that the disease is not so likely to recur if checked with the following—

℞ Cinchoniæ sulph.......................... gr. xx,

Pulv. capsici.............................. } aa gr. v,

Pulv. ext. colocynth, c., ................... }

Pulv. iwecac, comp.......................... gr. x.

Ft. chart. No. x.

M. Sig. One every two or three hours; the last one to be taken two hours before the expected chill.

This prescription should be given in wafers or capsules. A purgative is hardly ever required, as the colocynth combined generally moves the bowels sufficiently; and another great advantage of this prescription is its small cost, which is quite an item to those physicians who, like myself, have to furnish their own drugs; and, last, is its certainty; it will not fail as often as sulphate of quinine.

I have been fighting malarial diseases in a very malarious vicinity for the last ten years, and where the disease shows a stubborn tendency to return, I prescribe the following bitter—which is bitter sure enough, but will do the work if taken according to directions:

℞ Sulph. cinchonidia or quiniæ........................ ʒij,
Ferri cit.................................... ʒiij,
Spiritus frumenti.............................. Oij.
M. Sig. Tablespoonful before each meal.

This amount is generally sufficient to eradicate any case of simple ague, and has a tendency to give the patient an appetite, and remove that sallow and anæmic condition so universally found in those in whom ague has become chronic. I do not think there is any likelihood of the patient having an appetite created for stimulants, from this prescription. This is rather an expensive treatment when arsenic is so cheap, but arsenic is not so certain, nor does it meet all the indications so well; but a solution of arseniate of potassa, week about with the above, is very good treatment.

This article is a great deal longer than I expected it to be when I commenced. But if you think it would be of advantage to S. S. F., or any of the profession, publish it; otherwise, file it away in the waste basket.—A. P. LEVICK, M. D., *in Med. and Surg. Rep.*

**Antiseptic Inhalation.**—There can be no doubt of the benefit derived from antiseptic inhalation in phthisis. This benefit is chiefly in quieting the cough, and doing away with enough mixtures which are so sure to disturb the digestion. As a working formula Dr. J. G. Sinclair Coghill, of the National Hospital for Consumption (British Medical Journal, May 28, 1881), uses the following:—

℞. Tinct. iodi ether,<br>

Acidi carbolici.......................... aa ʒij<br>

Creasoti (or thymoli)..................... ʒj<br>

Alcoholis.......................... ad ℥j. M.

When the cough is urgent, chloroform or ether may be added at discretion. This is to be used in a respirator.—*Medical and Surgical Reporter.*

**Hop Bitters.**—We find in an exchange the following, given as the composition of this nostrum:—

℞. Tincture of hops.......................... f. ℥ ss<br>

Tincture of buchu.......................... ʒ iij<br>

Tincture of senega.......................... ʒ iij<br>

Podophyllin, gr. j dissolved in spirits of wine, ℥ ss<br>

Tincture of cochineal..................... gtt. xx<br>

Distilled water.......................... ad ℥ xvj. M.

The cost of these ingredients, based on prices quoted, amounts to about ten cents. The selling price of the mixture is one dollar.—*Medical and Surgical Reporter.*

**For Dyspepsia.**—

℞. Sodii bicarbonatis.......................... ʒ ij<br>

Tincturæ nucis vomicæ...................... fʒ ij<br>

Tincturæ gentian comp.,<br>

Tincturæ rhei simplic................... aa f℥ ij M.

Sig.—Shake well. Teaspoonful three times a day.—*Dr. H. Engal.*

**A Combined Stomachic and Laxative.**—This will be found serviceable in indigestion from stomachic debility and biliousness:

℞. Ext. boldo fluidi.
Ext. rhamnus pursh. fluidi.
Ext. cuonymi purp. fluidi.
Glycerinæ,......................................aa ℥ss.
Ext. nucis vomicæ fluidi, ..............................ʒ j.
Aq. cinnamomi,......................................℥ j.

M. Sig.—A teaspoonful three times a day.—*Thur. Gazette.*

**For Gonorrhœa.**—Here is another candidate for the $100 prize. It is submitted by Dr. J. H. Buchanan, White Bluff, Tenn.:

After preparing the system as may be required, give the following internally for the first stage:

℞. Ext. buchu fluidi.
Spts. etheris netrosi.................................aa ℥ ij.

M. Sig.—A teaspoonful three times a day.

℞. Ext. hydrastis canadensis.............................fl. ℥j.

Aquæ ros.......................................... ℥jv.

M. Sig.—Inject twice a day

For the second stage of inflammation: Continue the first prescription internally and inject the following:

℞. Quiniæ sulph. gr. ij
Acidi sulph. dil. gtt. viij.
Aquæ ros..........................................℥ j.

M. Sig.—Half of this mixture to be used at an injection.

In the third stage give internally:

℞. Tr. cantharidis......................................℥ j.
Quiniæ sulph........................................℥ss.
Tr. ferri chloridi.....................................℥ ij.
Acidi sulph. dil. gtt. xxx.
Aq. dist...........................................℥viij.

M. Sig.—A tablespoonful three times a day.

Employ mild astringent injections.

**Purgative Pill.**—For one pill:

℞. Res. podophyllin.

Aloin...........................................aa gr. ¼.

Ext. hyoscyami,....................................gr. ss. M,

The latter is an excellent cholagogue, operating without pain or nausea.—*Therapeutic Gazette.*

